# From Angiotensin II to Cyclic Peptides and Angiotensin Receptor Blockers (ARBs): Perspectives of ARBs in COVID-19 Therapy

**DOI:** 10.3390/molecules26030618

**Published:** 2021-01-25

**Authors:** John Matsoukas, Vasso Apostolopoulos, Anthony Zulli, Graham Moore, Konstantinos Kelaidonis, Kalliopi Moschovou, Thomas Mavromoustakos

**Affiliations:** 1 Institute for Health and Sport, Victoria University, Melbourne, VIC 3030, Australia; vasso.apotolopoulos@vu.edu.com (V.A.); Anthony.Zulli@vu.edu.au (A.Z.); 2 Department of Physiology and Pharmacology, Cumming School of Medicine, University of Calgary, Calgary, AB T2N 4N1, Canada; 3 NewDrug, P.C., Patras Science Park, 26504 Patras, Greece; k.kelaidonis@gmail.com; 4 Pepmetics Inc., 772 Murphy Place, Victoria, BC V8Y 3H4, Canada; mooregj@shaw.ca; 5 Department of Chemistry, National and Kapodistrian University of Athens, Zographou, 15784 Athens, Greece; kmoschovou@chem.uoa.gr (K.M.); tmavrom@chem.uoa.gr (T.M.)

**Keywords:** angiotensin II, RAS, cyclic peptides, sarmesin, sartans, mimetics, transdermal delivery, Covid 19, Sars-CoV-2

## Abstract

The octapeptide hormone angiotensin II is one of the most studied peptides with the aim of designing and synthesizing non-peptide mimetics for oral administration. To achieve this, cyclizations at different positions within the peptide molecule has been a useful strategy to define the active conformation. These studies on angiotensin II led to the discovery of Sarmesin, a type II angiotensin II antagonist, and the breakthrough non-peptide mimetic Losartan, the first in a series of sartans marketed as a new generation of anti-hypertensive drugs in the 1990s. Angiotensin II receptor blockers (ARBS) and angiotensin I converting enzyme inhibitors (ACEI) were recently reported to protect hypertensive patients infected with SARS-CoV-2. The renin–angiotensin system (RAS) inhibitors reduce excess angiotensin II and increase antagonist heptapeptides alamandine and aspamandine which counterbalance angiotensin II and maintain homeostasis and vasodilation.

## 1. Introduction

### 1.1. From Angiotensin II to Losartan and ARBS

Pioneer research on the renin–angiotensin system (RAS) has resulted in the discovery of the first orally active non-peptide angiotensin II receptor antagonist losartan (DUP753) [1,2]. This discovery was followed by related non-peptide angiotensin receptor antagonists (ARBs) marketed in the treatment of hypertension and representing a breakthrough new generation of antihypertensives. An overview of the pharmacokinetic parameters for seven angiotensin receptor antagonists (losartan, valsartan, irbesartan, candesartan, telmisartan, eprosartan, olmesartan) presented their properties [3]. Our group has been involved for years in the investigation of the mechanism through which angiotensin II exerts its hypertensive action. This research has led to new insights revealing the bioactive conformation of the hormone and the unique way angiotensin II is approaching its receptors [4,5]. In particular, these studies revealed a ring cluster conformation and a charge relay system between the aromatic amino acids Tyr, His, and Phe of the peptide molecule analogous to the charge relay system observed in serine proteases [6,7]. Cyclization of the angiotensin II peptide molecule connecting amino acids at different positions within the sequence was a novel strategy to confirm the bioactive ring cluster conformation suggested by NMR/NOE studies and the mechanism of ANGII action suggested by SAR and fluorescence studies.

### 1.2. From Linear to Cyclic Peptides

**Figure 1:**
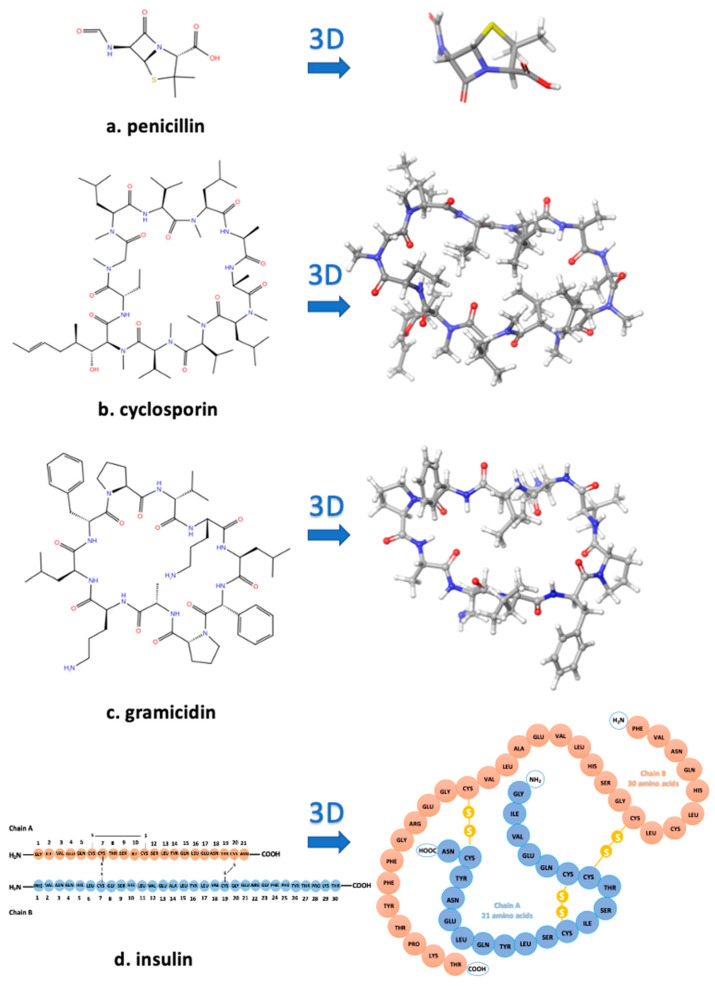
The 2D and 3D structures of well-known cyclic peptides. (**a**) Penicillin is one of the most powerful antibiotics. (**b**) Cyclosporin is a natural product used as an immunosuppressant medication. (**c**) Gramicidin exhibits strong antibiotic activity. (**d**) Insulin is a hormone that plays a key role in the regulation of blood glucose levels, and its deficiency causes diabetes.

Cyclic peptides constitute a class of compounds that were used in the treatment of certain diseases. Examples of such well-known cyclic peptides are insulin, penicillin, cyclosporin, and gramicidin S. Cyclic peptides, compared to linear peptides, show greater potential as therapeutic agents due to their increased chemical and enzymatic stability, receptor selectively, and improved pharmacodynamic properties (Figure 1). In our peptide research, cyclization of peptides is a key step towards the design of non-peptide mimetics which is the final target [8]. Our research group was the first, worldwide, to synthesize cyclic analogs of important peptides such as angiotensin II (for hypertension), myelin epitope peptides (for multiple sclerosis), gonadotropin releasing hormone (for infertility and cancer), and thrombin receptor activating peptides (for angiogenesis and cancer) [4]. Another way of transforming peptides to peptide mimetics is by conjugating peptides to sugars, such as mannan, used as antigen carriers in cancer and in multiple sclerosis research [5,6,7,8,9]. Figure 1a–d shows the 2D and 3D structures of well-known cyclic peptides penicillin, cyclosporin, gramicidin, and insulin.

## 2. The Strategy to Build Non-Peptide Mimetics

**Figure 2:**
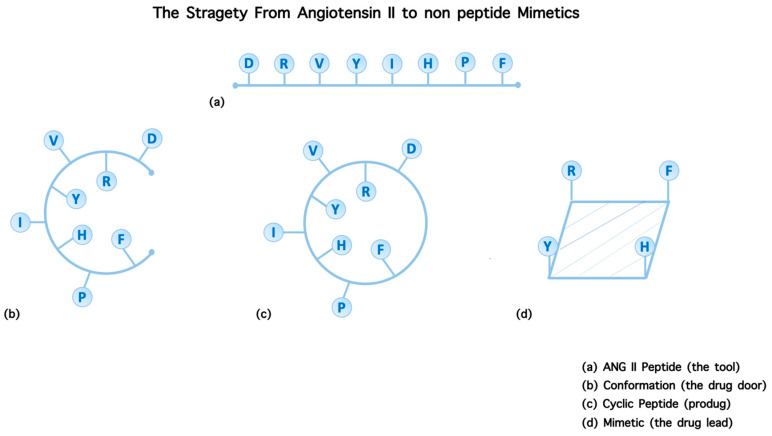
Steps of the strategy to design a non-peptide ANG II mimetic. (**a**) ANG II peptide (the tool)—SAR studies to identify the critical amino acids. (**b**) Conformation (the drug door)—NMR/NOE studies to approach the bioactive conformation. (**c**) Cyclic peptide (prodrug) —cyclization at positions least important for activity. (**d**) Mimetic (the drug lead)—synthesis of a non-peptide mimetic based on critical amino acids.

Synthesis of cyclic peptides, in our studies, was pursued as an intermediate step towards constructing orally active non-peptide mimetics. Cyclic peptides, compared to linear counterparts, present many advantages in terms of stability, binding, selectivity, and activity. The limited stability of peptides, due to hydrolysis of amide bonds, severely restricts their medical and industrial application. Therefore, the engineering of stable peptide moieties which are the cyclic counterparts and non-peptide mimetics is of the highest importance. Cyclic peptides are the bridges between linear peptides and their non-peptide mimetics and cyclization is a strategy to build non-peptide mimetics. Furthermore, cyclizations were a way to define and lock the active conformation of the peptide. Steps towards mimetics are: (I) identification of critical amino acids of the peptide sequence through alanine screening and SAR studies; (II) approaching the bioactive peptide conformation through in silico, modeling, crystallography, and NMR studies (III); cyclization of the peptide through amide bonds between amino acids at positions least important for activity; (IV) the design and synthesis of non-peptide mimetics is based on the knowledge of the active pharmacophoric side chain of amino acids critical for binding and triggering activity. The pharmacophoric side chains are attached to an organic scaffold and the whole moiety acts as a lead non-peptide mimetic to be administered orally if it meets the pharmacological criteria [4]. Figure 2 shows the strategic steps from peptides to non-peptide mimetics.

## 3. Rational Design of Potent Cyclic Peptides Based on SAR (Structure–Activity Relationship) and NMR Studies

**Figure 3:**
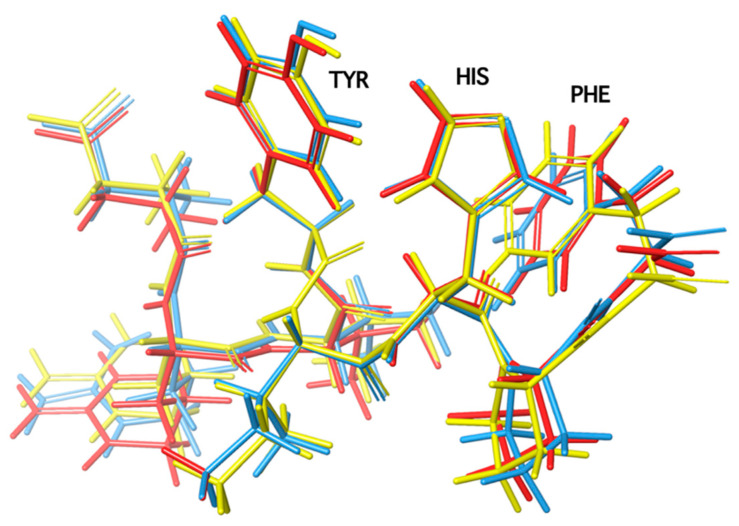
Putative bioactive conformation of angiotensin II based on NMR spectroscopy and molecular dynamics.

The design of potent cyclic analogs for bioactive peptides requires the knowledge of the role of each amino acid residue, especially those involved in receptor activation, as well as the active conformation of the peptide. Structure–activity studies have shown the importance of the three aromatic amino acids Tyr, His, and Phe and the C-terminal carboxylate for activity. In order for cyclic analogs to retain the activity of the linear peptide, cyclization should occur at residue positions which are the least important for activity with retention of the bioactive conformation. The conformation of peptides is deduced from nuclear magnetic resonance techniques, such as two-dimensional NMR ROESY, NOESY, COSY, TOCSY, and 1-D NOE. These techniques shed light on the spatial place of amino acid side chains within the peptide molecule. For example, in angiotensin II, residues Val and Ile at positions 3 and 5 are the least important for triggering ANGII receptors. NMR studies with ROESY, NOESY, and 1D-NOE (nuclear overhauser effects) techniques revealed a cluster of the three aromatic rings Tyr, His, and Phe, which together with the C terminal carboxylate are important for binding and activity of the peptide hormone. Therefore, cyclization between residues at the 3 and 5 positions affords a cyclic product that retains activity as the important pharmacophoric residues Tyr, His, and Phe remain intact with their side chains closely spaced, a requirement for ring cluster and for activity in linear angiotensin II. Positions Val3, Ile5, and Pro7 serve the proper backbone orientation whilst Arg at position 2 stabilizes the tyrosine anion, which triggers the activity of the peptide hormone [10,11,12,13]. Figure 3 shows the conformation of angiotensin II obtained from 1D-NOE, NOESY, and ROESY data and fluorescence lifetime studies [10,11,12,13].

## 4. The Renin–Angiotensin System (RAS) Regulates Vasodilation

**Figure 4:**
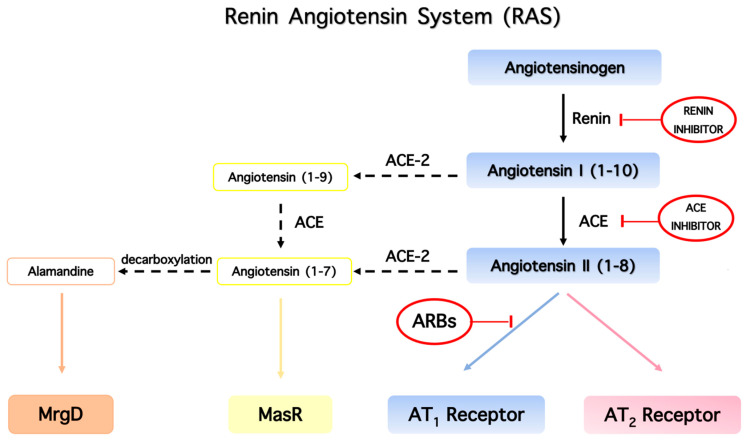
The main components of the renin–angiotensin system are the counterbalancing axes: ACE/ANGII/AT1R (vasoconstriction), ACE2/A(1–7)/AT2R/MasR (vasorelaxation), and ACE2/alamandine/MrgD (vasorelaxation).

The renin–angiotensin system (RAS) has been identified in various tissues including heart, brain, kidneys, and vessel walls, and is characterized by a sequence of enzymatic reactions. The RAS system is the most important system regulating blood pressure. Angiotensinogen formed in the liver is cleaved by the enzyme renin to form the inactive decapeptide angiotensin I (ANG I), which is further cleaved by angiotensin-converting enzyme (ACE1) to form the octapeptide hormone (Asp-Arg-Val-Tyr-Ile-His-Pro-Phe) angiotensin II (ANG II). The RAS system plays a pivotal role in cardiovascular homeostasis and the regulation of blood pressure. In addition, it was found that ACE2 receptors are an important element of the RAS system, as it converts ANG II to heptapeptides alamandine and aspamandine, which are beneficial for vasodilation and homeostasis [14,15,16] (Figure 2). Figure 4 shows the main components of the renin–angiotensin system that are the counterbalancing axes: vasoconstriction axis (ACE/Ang II/AT1R), vasorelaxation axis (ACE2/A(1–7)/AT2R/MasR), and vasorelaxation axis (ACE2/alamandine/MRgD).

## 5. The Role of Phenylalanine for Hypertensive Activity of Angiotensin

**Figure 5:**
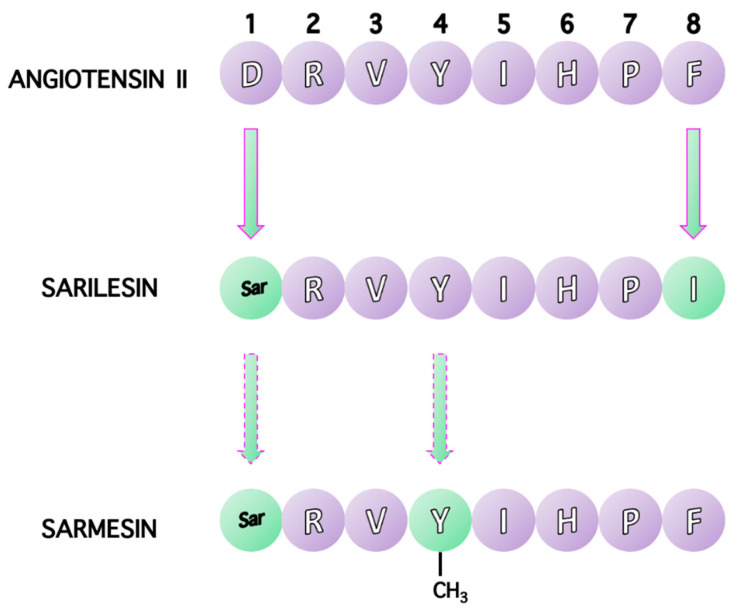
The peptide sequences of angiotensin II (Asp-Arg-Val-Tyr-Ile-His-Pro-Phe), sarilesin [Sar^1^, Ile^8^] ANGII, and sarmesin [Sar^1^, Tyr (OMe)^4^] ANGII. Phenylalanine at position 8 and tyrosine at position 4 are crucial for triggering hypertensive activity.

Due to the biological importance of linear octapeptide ANG II, a large number of ANG II analogs were synthesized in order to establish the roles of ANG II residues. Structure-activity studies (SAR) illustrated the importance of the C-terminal aromatic residue phenylalanine (Phe) as well as the C-terminal carboxylate for agonist activity. Replacement of the aromatic residue Phe at position 8 with an aliphatic one, such as Ile, results in an antagonist, (Sar1, Ile8) ANG II (sarilesin) (H-Sar1-Arg-Val3-Tyr-Ile5-His-Pro7-Ile8-OH). Essential π*-π* interactions between Tyr and Phe rings observed in ANG II and its superagonist Sar1 ANG II are missing in sarilesin. Furthermore, deletion of the aromatic amino acid phenylalanine at position 8 results in the vasodilator heptapeptides, aspamandine [ANG1–7] and upon decarboxylation alamandine ANG [Ala1-7], which counterbalance the toxic ANG II in the RAS system [14,15,16]. Figure 5 shows amino acid sequences for angiotensin II, sarilesin and sarmesin.

## 6. Charge Relay System (CRS) and the Role of Tyrosine Hydroxyl in Triggering Activity of Angiotensin II

**Figure 6:**
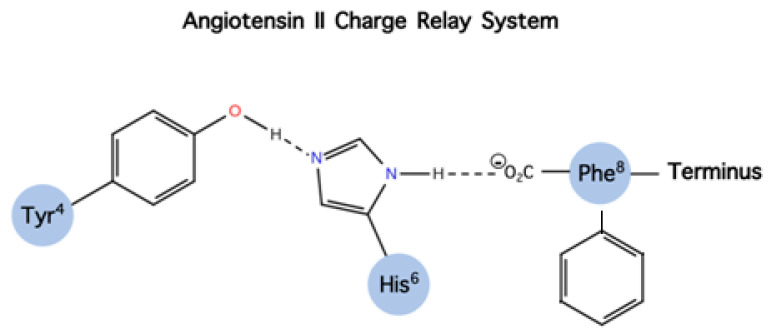
Tyr, His, and Phe aromatic side chains and c-terminal carboxylate are intra connected to create a cyclic compact structure that triggers activity through Tyr hydroxylate.

**Figure 7:**
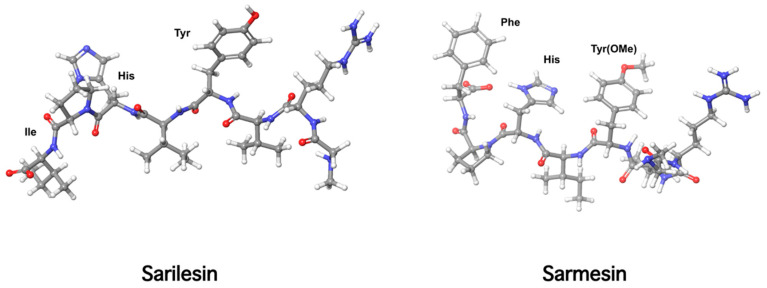
3D structures of sarilesin and sarmesin.

Our laboratory has been engaged for many years in the conformational analysis of ANG II, its superagonist [Sar1] ANG II, and structurally similar peptide agonists and antagonists. These studies led to the discovery of sarmesin, a type II antagonist of angiotensin II, where tyrosine hydroxyl is methylated [17,18,19,20]. Methylation of the tyrosine hydroxyl group eliminates activity revealing the importance of a tyrosinate negative charge for potency. These studies led, furthermore, to the hypothesis of a charge relay system involving the triad tyrosine, histidine, and phenylalanine carboxylate closely spaced in a ring cluster conformation, where the tyrosinate negative charge triggers activity. The charge relay system creates a cyclic structure within the angiotensin II molecule which at the receptor level operates through the tyrosine hydroxylate to trigger activity. Methylation of tyrosine hydroxyl in position 4, as in sarmesin, disrupts the charge relay system and eliminates the hypertensive activity of angiotensin II. This hypothesis was confirmed by fluorescence studies of angiotensin II analogs in receptor-simulating environments [21,22] (Figure 6 and Figure 7). Figure 6 shows the angiotensin II relay system and Figure 7 shows models of sarilesin and sarmesin.

## 7. Cyclic Angiotensin II Analogues Confirm the Ring Cluster Conformation of ANG II

**Figure 8:**
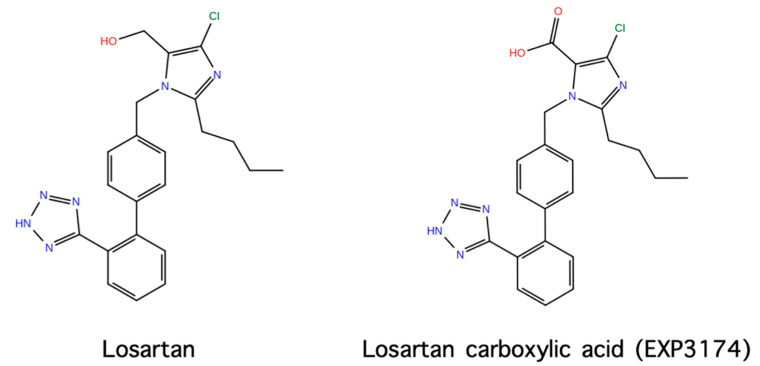
Losartan and the active metabolite losartan carboxylic acid.

Based on the above studies, we designed and synthesized novel amide linked ANG II cyclic analogs: cyclo (3,5) (Sar1, Lys3, Glu5) ANG II and cyclo (3,5) (Sar1, Lys3, Glu5, Ile8) ANG II to confirm the necessity of the three aromatic rings for activity and to define the active conformation (23–25). Cyclization was achieved by forming an amide-linkage between the –NH2 and –COOH side chain groups of Lys and Glu residues at positions 3 and 5, respectively, which are the least important for activity [23,24,25]. In particular, the constrained amide linked cyclo (3,5) (Sar1, Lys3, Glu5) ANG II analog which retains the three aromatic rings and the c-terminal carboxylate was found to be active in both ex vivo and in vivo experiments. As expected, cyclo (3,5) (Sar1, Lys3, Glu5, Ile8), where aromatic phenylalanine was substituted by aliphatic isoleucine at position 8, did not preserve hypertensive action. This analogue was found to be a potent ANG II antagonist and reduced ANG II dependent hypertension in a dose-related manner. Based on losartan, sarmesin, and our ring cluster and charge relay system conformation, we designed and synthesized angiotensin II receptor blockers by rotation from C-2 to C-5 of the alkyl chain on the imidazole ring. This rotation resulted in losartan derivatives of similar activity to losartan. A novel synthetic strategy was applied to accelerate synthetic steps for losartan V8 and losartan V8 analogs [26,27,28], compared to the initial synthesis [29]. Figure 8 shows the structures of losartan and active metabolite losartan carboxylic acid (EXP 3174).

## 8. From Potent Cyclic (3,5) Angiotensin II to Losartan and ARBS—A Simulation Study

**Figure 9:**
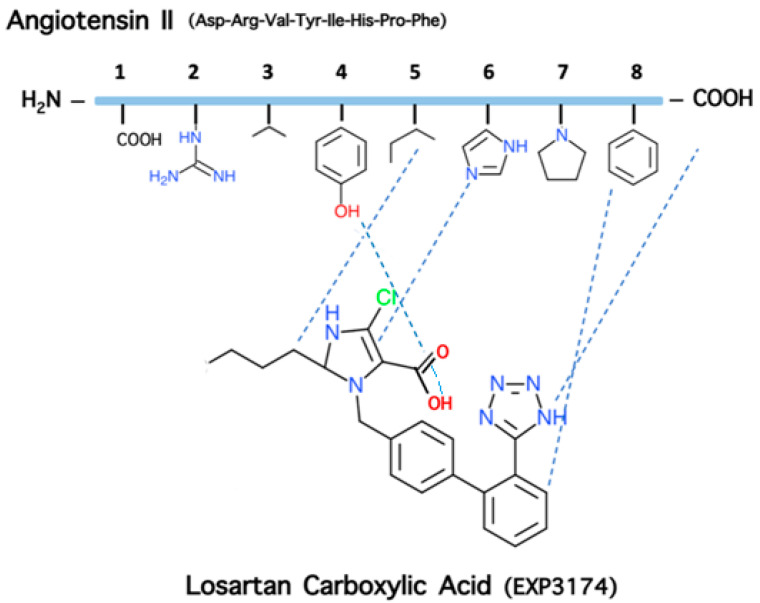
Correlation of pharmacophoric side chains of angiotensin II with essential groups in losartan carboxylic acid (EXP 3174).

The activity of the constrained amide linked cyclo (3,5) (Sar^1^, Lys^3^, Gly^5^) ANG II analog preserving the three critical aromatic amino acids (Tyr, His, Phe) and the c-terminal carboxylate of ANG II together with the NMR nuclear overhauser effect (NOE) findings suggested a cluster of the aromatic side chain in angiotensin II and a charge relay system (CRS) connecting the three aromatic rings (tyrosinate, imidazole, and phenyl) and the c-terminal carboxylate (Figure 3). The proximity of the three aromatic side chains of Tyr, His, and Phe and of the c-terminal carboxylate—critical structural features for activity—suggests the design of a non-peptide ANG II mimetic which should contain the pharmacophoric groups of the ANGII peptide. This assumption leads to a non-peptide molecule mimicking the native peptide and capable of competing with angiotensin II for binding to the receptors, and this indeed happens as in the case of losartan and ARBS. In particular, based on the active conformation of angiotensin II, the Ang II non-peptide mimetic should contain the following features: (i) an imidazole ring as scaffold corresponding to the imidazole side chain of histidine at position 6 of Ang II; (ii) a phenyl ring, present in the attached biphenyl on the imidazole scaffold, corresponding to the side chain of phenylalanine at position 8 of Ang II; (iii) a negatively charged group as the carboxylate attached in the imidazole ring of losartan carboxylic acid (EXP 3174), corresponding to the tyrosinate at position 4 of the Ang II peptide; (iv) a negative charge on a phenyl ring of the mimetic which could be the highly acidic tetrazole group in the attached biphenyl, corresponding to the c-terminal carboxylate of angiotensin II; (V) an aliphatic chain which is the butyl chain on the imidazole ring of the mimetic, corresponding to the aliphatic side chain of leucine at position 5 of Ang II in losartan carboxylic acid (EXP3174) which is the active metabolite of losartan triggering activity, confirming the essential role of the negative charge in the carboxylate anion for higher affinity compared to the hydroxylate anion in losartan [26,27,28]. Affinity is enhanced due to the electronegative chlorine atom at position 4 of the imidazole ring which stabilizes the carboxylate anion. Figure 9 shows the correlation of pharmacophoric side chains of angiotensin II with essential groups in losartan carboxylic acid (EXP 3174).

## 9. Losartan Carboxylic Acid (EXP 3174) is a Stronger Binder Compared to Losartan

**Figure 10:**
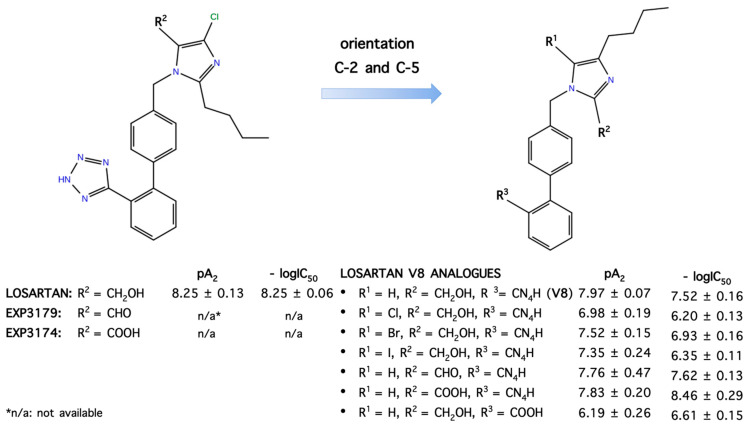
Structures and activities of losartan and losartan V8 analogs.

Overall, the critical structural features of angiotensin II are depicted clearly in the structure of the polyaromatic losartan carboxylic acid, the active metabolite of losartan, which contains all pharmacophoric groups of angiotensin II and the two negative charges (phenyl tetrazolate and imidazole carboxylate), a requirement for affinity. The tetrazole group is highly acidic, as its tetrazolate anion is stabilized by the resonance in the aromatic tetrazole ring. The imidazole carboxyl group in active metabolite is highly acidic, as its carboxylate anion is stabilized by the resonance in the aromatic imidazole ring aided by the highly electronegative chlorine atom at position C2 of the imidazole ring. Chlorine is a strong electron acceptor atom stabilizing the carboxylate anion. Our angiotensin receptor antagonists with reversed positioning of the alkyl and methylene hydroxyl groups on the imidazole ring were designed based on our ring cluster conformation, the cyclization findings, and on the sarmesin structure which revealed the importance of the tyrosine hydroxyl group for activity [19]. These compounds were evaluated for binding to human AT1 receptor and for ANG II antagonism in vitro on isolated rat uterus. Among them, 5-butyl-1-[[20-(2H-tetrazol-5-yl)biphenyl-4-yl]methyl]imidazole-2-carboxylic acid (losartan analogue V8COOH) exhibited higher binding affinity compared to the other analogues tested (−log IC_50_ = 8.46). The latter analog was also found to be the most active in the rat uterotonic test (pA2 = 7.83). Importantly, the binding affinity of the losartan analog V8COOH was higher than that of losartan (−log IC_50_ = 8.25) indicating the importance of the carboxyl group at the C-2 position. Our receptor antagonist (losartan analog V8COOH), the counterpart of losartan carboxylic acid (EXP3174), displayed higher binding affinity (−log IC_50_ = 8.46) compared to losartan (−log IC_50_ = 8.25), indicating that a carboxylate anion binds stronger compared to the methylene hydroxyl group (−log IC_50_ = 8.25). Angiotensin II receptor binding studies and simulation studies between angiotensin II and losartan carboxylic acid (EXP3174) provide insights for the ligand–receptor interaction and for the relationship between the pharmacophoric groups of the two molecules as depicted in Figure 9. [30,31]. Figure 10 shows structures of losartan analogs synthesized in our studies compared to losartan and its metabolites EXP 4179 and EXP 3174.

## 10. Receptor Desensitization by Angiotensin II Antagonist Sarilesin

Two types of angiotensin peptide antagonists exist: type I is characterized by powerful receptor desensitizing effects resulting in a long duration of action (e.g., sarilesin or (Sar^1^, Ile^8^) angiotensin II), whereas type II are reversible competitive antagonists (e.g., sarmesin or [Sar^1^, Tyr(Me)4]angiotensin II [32]. When the desensitizing antagonist sarilesin is methylated at its Tyr hydroxyl it is converted to a competitive antagonist, illustrating that the tyrosine anion (formed by the charge relay mechanism) is responsible for receptor desensitization (in addition to having a role in receptor activation). In parallel to the situation with the peptides, the conversion of the hydroxymethyl group of losartan (competitor) to the carboxylate anion metabolite EXP 3174 (desensitizer) accounts for the long duration of action of ARBs, [33]. Receptor activation (A) and receptor desensitization (D) effects may involve two different binding modes on the receptor [32]. Since angiotensin itself at high doses can desensitize its own receptors (an overkill defense mechanism used by many other ligands), this could imply a slightly higher affinity for the A site (resulting in receptor dimerization and amplification of the G protein coupled response) compared to the D site (preventing receptor dimerization and decoupling G protein for an extended period of receptor lockdown) [32,33]. Interestingly, crystallography studies have implicated the presence of a charge relay system analogous to that shown in Figure 6, in which the carboxylate/ imidazole of the ARB olmesartan links to the hydroxyl of Tyr35 of the receptor [34] in its D format. Although the mechanism of receptor desensitization is not well understood, it could involve decoupling of receptors from the signaling mechanism, possibly by inducing a long-term conformation change in the receptor (which is ultimately reversible). Like ARBs, many long-acting therapeutically useful drugs work by this important mechanism. Furthermore, extensive structure–activity studies on sarilesin and sarmesin revealed the role of the N-terminal in the overall conformation of the angiotensin II agonist and antagonist peptides. As is evident from the data, the requirement for a single alkyl substitution at the N-terminus of sarmesin is obligatory for its unique actions [34,35]. These data emphasize the stringent and discriminating structural requirements in the N-terminal domain of sarmesin that endow this analog with its antagonist properties and suggest the presence of defined steric constraints in this region of the molecule during receptor blockade [35,36,37].

## 11. Bis Alkylated Arbs Display Strong Affinity and Antagonist Activity

**Figure 11:**
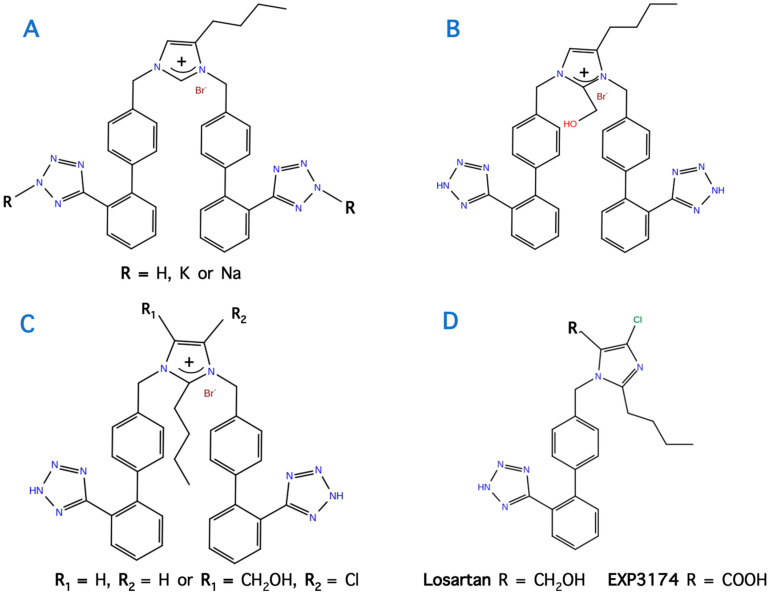
Bis biphenyl alkylation of the imidazole ring of losartan V8 (analogs **A**,**B**) and losartan (analog **C**) by a convenient and cost-effective strategy.

**Figure 12:**
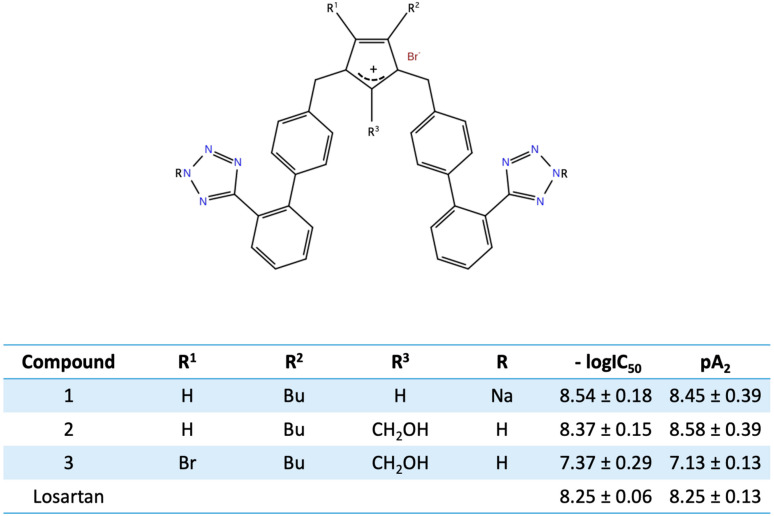
Structures and biological activities of bis alkylated losartan (**1**) and losartan V8 analogs (**2**, **3**).

In our studies for new angiotensin II receptor antagonists, we achieved bis alkylation of the imidazole ring by a convenient and cost-effective synthesis strategy [38,39,40]. A series of symmetrically bis-substituted imidazole analogs bearing at the N-1 and N-3 two biphenyl moieties ortho substituted either with tetrazole or carboxylate functional groups was designed based on docking studies and utilizing for the first time an extra hydrophobic binding cleft of the AT1 receptor. The synthesized analogs were evaluated for their in vitro antagonistic activities (pA2 values) and binding affinities (−logIC_50_ values) to the angiotensin II AT1 receptor. Among them, the potassium (−logIC_50_ = 9.04) and the sodium (−logIC_50_ = 8.54) salts of 4-butyl-N,N’-bis{[20-(2H-tetrazol-5-yl)]biphenyl-4-yl]methyl}imidazolium bromide as well as its free acid 11 (−logIC_50_ = 9.46) and the 4-butyl-2-hydroxymethyl-N,N0-bis{[20-(2H-tetrazol-5-yl)biphenyl-4yl]methyl}imidazolium bromide (−logIC_50_ = 8.37, pA2 = 8.58) showed high binding affinity to the AT1 receptor and high antagonistic activity [39,40]. These results may contribute to the discovery and development of a new class of biologically active molecules through bis-alkylation of the imidazole ring by a convenient and cost-effective synthetic strategy. Figure 11 and Figure 12 show structures after bis biphenyl alkylation of the imidazole ring in both losartan and our losartan V8 analog. Bis alkylated analogs were synthesized with a convenient and cost-effective strategy [38,39,40].

## 12. Perspectives of Arbs in Transdermal Treatment of Hypertension

ARBs can also be administered transdermally, although not yet in practice as an accepted therapy. We have developed a transdermal delivery method for ARBs (losartan and valsartan) and determined the anti-hypertensive effects of the transdermal delivery patch in a steady-state release [41]. Transdermal drug delivery is a novel approach for the administration of ARBs [42,43]. This method offers several important pharmacological advantages over conventional dosage forms, such as avoidance of the first-pass metabolism by the liver, minimizing pain, controlled release of a drug, and prolonged duration [43,44,45]. The majority of drugs that are administered orally have reduced efficacy due to first-pass metabolism, as well as to related side effects. For several diseases such as diabetes (insulin), multiple sclerosis (interferons), and cancer (taxol), drugs taken by injections must be administered in a painless and effective manner, which is the transdermal or nasal method. In this regard, transdermal delivery of drugs including ARBs can bypass these issues. For example, new transdermal peptides are pursued for multiple sclerosis treatment [46]. Thus, controlled release systems have emerged to overcome the disadvantages of the oral or injection route and ARBs meet the criteria to be administered transdermally. In our studies, ARBs were able to penetrate the stratum corneum (SC), a barrier to the absorption of such drugs [47,48,49,50]. Specific drug characteristics are required for efficacy, including molecular size and weight less than 500 Da, significant lipophilicity, efficacy in low plasma concentration, and a high degree of stability, which ARBs possess. To enhance transdermal absorption of ARBs, drug derivatives, prodrugs, drug saturated systems, and physical and chemical enhancers that facilitate the permeation of the drug through the stratum corneum can be used [50,51,52].

## 13. Perspectives of ARBs in COVID-19 Therapy

The ACE2 enzyme has been identified to be the binding site of SARS-CoV-2 virus and controversy rose if the RAS system could be a therapeutic target, as an option to treat the disease by blocking the binding [14]. Recent findings have shown that ARBs have protective effects in hypertensive patients infected by SARS-CoV-2 [53,54,55,56,57,58,59]. This article elaborates on the mechanism which triggers the hypertensive activity of angiotensin II and on the idea of considering ARBs as a tentative treatment for SARS-CoV-2 infections. Furthermore, it proposes a research direction using ARBs (drug repurposing) based on the recent findings. The idea is to block the ACE2 receptor which serves as the binding site for SARS-CoV-2 while preserving the integrity of the enzyme. In particular, the strategy is to enhance the ACE2 enzyme which converts toxic ANG II to the protective antagonist heptapeptides ANG(1–7) and alamandine, maintaining homeostasis and at the same time blocking the entry of SARS-CoV-2 to ACE2 [14]. This is possible with ACE2 activators like DIZE [14] and with non-peptide mimetics of Ang II which upregulate ACE2, producing the two heptapeptides that counterbalance destructive ANG II and are beneficial for vasodilation. It is very likely that uncontrollable release of pathogenic ANG II may be one of the reasons for the storm of cytokines and pneumonia in COVID-19. In particular, positive effects include ACE2 receptor blockade, disabling viral entry into the heart and lungs, and an overall decrease in inflammation secondary to ACE1/ARB. The well-studied reduction in mortality conferred by ACE1/ARB use and the beneficial effects for patients with diabetes, chronic kidney disease, and proteinuria or albuminuria currently outweigh the theoretical risks [58]. Our group has studied extensively the RAS system and these studies led to the rational design and synthesis of angiotensin receptor blockers (ARBs) which could block COVID-19 pathogenicity. Our in silico studies with our ARBs are in line with the recent studies on losartan, which show a high affinity for spike protease and attenuate SARS-CoV-2 [59]. These studies on the renin–angiotensin system showed that over activation of AT1R by hyper-acute excess of angiotensin II due to acute downregulation of ACE2 by SARS-CoV-2 explain the mechanism of a cytokine storm in COVID-19. The in silico studies suggest that losartan and ARBs are a promising COVID-19 treatment. Previous clinical studies indicate a safe and protective role of the RAS inhibitors [57,58].

In summary, it has become clear recently from clinical findings that ARBs have a protective effect in hypertensive patients with COVID-19. ARBs play a positive role in protecting ACE2 from the entry of SARS-CoV-2 virus, since ARBs, ACE2, and COVID-19 are evidently related. This relationship between RAS inhibitors and COVID-19 has already been reported [60,61,62,63,64,65,66,67,68,69,70]. The important function of ACE2 is its upregulation by the components of the A(1-7)/AT2R/MasR axis generated by peptides and non-peptide agonists [59]. This function is generally acknowledged as a pivotal link between ACE2 deficiency and SARS-CoV-2 infection [61,62,63,64,65,66,67,68,69]. On the contrary, overexpression of ACE2 is linked with protection from SARS-CoV-2 [14,16,63]. Accumulated data presented in the cited articles show that RAS inhibitors improve clinical outcomes of COVID-19 patients with hypertension [66]. These findings propagate the use of ARBs in hypertension-related to COVID-19 diseases [64]. A large clinical trial (BRACE CORONA TRIAL) among patients hospitalized with COVID-19 infection and receiving chronic ACEI/ARBs is currently underway [69].

## 14. Conclusions

ARBs have been reported in comprehensive studies to affect the renin–angiotensin system (RAS) by upregulating the ACE2 enzyme more than other drugs prescribed for hypertension. This is of particular importance since ACE2 is the entry site of SARS-CoV-2 in the nasopharynx, lung, and cardiac cells. ARBs reduce pathogenic angiotensin II and increase beneficial heptapeptides, A(1–7) (aspamandine) and alamandine in the RAS equilibrium of angiotensin peptides. ARBs present the potential to positively affect the course of many heart diseases. The size, polarity, charges, and receptor selectivity render them ideal drugs to keep homeostasis and it seems to be a tentative therapeutic for SARS-CoV-2 infection. It is worth pursuing further studies to investigate and confirm ARBs as potential therapeutics for COVID-19 (drug repurposing).

## Abbreviations

**COVID-19:** Coronavirus Disease, **SARS-CoV-2**: Severe Acute Respiratory Syndrome Coronavirus 2, **NMR:** Nuclear Magnetic Resonance, **NOE:** Nuclear Overhauser Effect, **SAR:** Structure–Activity Relationship, **Alamandine:** [Ala^1^] Angiotensin (1–7), **Aspamandine:** [Asp^1^] Angiotensin (1–7), **AT1R:** Angiotensin II T1 Receptor, **RAS:** Renin–Angiotensin System, **ARBs:** Angiotensin Receptor Blockers, **CRS:** Charge Relay System, **SAR:** Structure Activity Relationship, **ANG II:** Angiotensin II, **A (1–7):** Angiotensin (1–7), **ACE:** Angiotensin-Converting Enzyme, **ROESY:** Rotating Overhauser Effect Spectroscopy, **NOESY:** Nuclear Overhauser Effect Spectroscopy, **COSY:** Correlated Spectroscopy, **TOCSY:** Total Correlated Spectroscopy, **1-D NOE:** One-Dimensional Nuclear Overhauser Effect.
